# A tool to evaluate proportionality and necessity in the use of restrictive practices in forensic mental health settings: the DRILL tool (Dundrum restriction, intrusion and liberty ladders)

**DOI:** 10.1186/s12888-020-02912-6

**Published:** 2020-10-23

**Authors:** Harry G. Kennedy, Ronan Mullaney, Paul McKenna, John Thompson, David Timmons, Pauline Gill, Owen P. O’Sullivan, Paul Braham, Dearbhla Duffy, Anthony Kearns, Sally Linehan, Damian Mohan, Stephen Monks, Lisa McLoughlin, Paul O’Connell, Conor O’Neill, Brenda Wright, Ken O’Reilly, Mary Davoren

**Affiliations:** 1 National Forensic Mental Health Service, Central Mental Hospital, Dundrum, Dublin 14, Ireland; 2 DUNDRUM Centre for Forensic Excellence, Department of Psychiatry, Trinity College Dublin, Dublin 2, Ireland; 3 Camlet Lodge Medium Secure Unit, North London Forensic Service, Chase Farm Hospital, Barnet Enfield and Haringey NHS MHT, London, UK; 4 Broadmoor High Security Hospital, Berkshire, UK

**Keywords:** Forensic psychiatry, Forensic, Violence, Seclusion, Restraint, Observational Study, Coercive

## Abstract

**Background:**

Prevention of violence due to severe mental disorders in psychiatric hospitals may require intrusive, restrictive and coercive therapeutic practices. Research concerning appropriate use of such interventions is limited by lack of a system for description and measurement. We set out to devise and validate a tool for clinicians and secure hospitals to assess necessity and proportionality between imminent violence and restrictive practices including de-escalation, seclusion, restraint, forced medication and others.

**Methods:**

In this retrospective observational cohort study, 28 patients on a 12 bed male admissions unit in a secure psychiatric hospital were assessed daily for six months. Data on adverse incidents were collected from case notes, incident registers and legal registers. Using the functional assessment sequence of antecedents, behaviours and consequences (A, B, C) we devised and applied a multivariate framework of structured professional assessment tools, common adverse incidents and preventive clinical interventions to develop a tool to analyse clinical practice. We validated by testing assumptions regarding the use of restrictive and intrusive practices in the prevention of violence in hospital. We aimed to provide a system for measuring contextual and individual factors contributing to adverse events and to assess whether the measured seriousness of threating and violent behaviours is proportionate to the degree of restrictive interventions used. General Estimating Equations tested preliminary models of contexts, decisions and pathways to interventions.

**Results:**

A system for measuring adverse behaviours and restrictive, intrusive interventions for prevention had good internal consistency. Interventions were proportionate to seriousness of harmful behaviours. A ‘Pareto’ group of patients (5/28) were responsible for the majority (80%) of adverse events, outcomes and interventions. The seriousness of the precipitating events correlated with the degree of restrictions utilised to safely manage or treat such behaviours.

**Conclusion:**

Observational scales can be used for restrictive, intrusive or coercive practices in psychiatry even though these involve interrelated complex sequences of interactions. The DRILL tool has been validated to assess the necessity and demonstrate proportionality of restrictive practices. This tool will be of benefit to services when reviewing practices internally, for mandatory external reviewing bodies and for future clinical research paradigms.

**Supplementary information:**

**Supplementary information** accompanies this paper at 10.1186/s12888-020-02912-6.

## Background

### Rationale

There is no validated psychological or psychiatric rating scale for use of restrictive and intrusive, coercive practices for the prevention of violence in psychiatric hospitals. No system exists for modelling and analysing how antecedents, behaviours, interventions and consequences can be quantified and studied. In the absence of such a system or paradigm, it is not surprising that these complex inter-relationships and the highly skilled clinical interventions involved are criticised for lack of a basis in evidence of effectiveness. We set out to devise and validate such a system. We hypothesised that a quantitative descriptive system could be designed for violent and disruptive behaviours and for preventive interventions including interventions that are restrictive or intrusive.

Admission and intensive care wards in forensic and other psychiatric hospitals are designed and operated to prevent violence and suicide as a necessary condition for providing effective treatments. In secure forensic hospitals, a priority is to maintain a therapeutically safe environment by preventing violence by patients against patients and others. This is accomplished mainly through the ‘standard model’ of stratified therapeutic security [1]. This places an emphasis on relational therapeutic security, the ratio of staff to patients and the quality of the therapeutic relationship between staff and patients. Various highly skilled interventions are deployed according to the model of care, the individual care and treatment plan, and day to day, minute to minute dynamic use of professional skills. Health technologies are also used, including the coordinated use of physical environmental design, equipment and technology [1, 2]. These interventions are complex, they involve difficult compromises between conflicting rights and principles, they can never achieve perfect outcomes and they require a tolerance of diverse approaches tailored to the individual while ensuring the personal safety and bodily integrity of others in the milieu.

Research in this area is sparse. Recent Cochrane reviews and systematic reviews found no studies that met inclusion criteria, much evidence for adverse effects and a lack of focus upon non-pharmacological methods for containment of violence or self-harm in people with serious mental illness [3–5].

Clinicians have a duty of care to ensure the safety of all patients in the therapeutic milieu. In many jurisdictions the safety and wellbeing of staff is also enshrined within law. Responsible clinicians therefore face a challenging ethical dilemma in balancing patient liberties against patient and staff rights not to be assaulted. A study involving 15,615 psychiatric patients within the state of California investigated the incidence of patient on patient assault in addition to patient on staff assault during the period 2011–2013 [6]. The findings were that 31.4% of psychiatric patients committed at least one violent assault during their hospitalisation. The number of patient-on-patient assaults was 10,958 and the number of patient-on-staff assaults was 8429. Of note, 1% of patients accounted for 28.7% of assaults suggesting that they were particularly refractory to medical and psychological interventions. The phenomenon of a minority of instances (here, patients) accounting for a majority of adverse events (here assaults) is well described in a variety of natural phenomena and managed processes, often attributed to Pareto [7]. The authors concluded that little is known about a) how to provide effective treatment or reduce the risk of assault carried out by these prolific, high risk patients and b) little is known about their motivations for carrying out the assaults which in some cases may be instrumental rather than expressive e.g. patients wishing to separate from other patients and staff due to a belief that that they will be harmed [8], or because of moralistic attitudes or cognitions [9, 10]. Or reactive violence related to impaired neurocognition and social cognition [11]. Consequently, the risk of being assaulted as a patient or a member of staff working in a forensic hospital is nontrivial [6].

It is not unusual to hear as a policy goal that services will achieve ‘zero’ seclusion or reduce seclusion or restraint by some arbitrary percentage per annum [12, 13]. These policies take no account of the likely compensatory increase in alternative interventions [14]. Forced rapid titration of medication by injection is more dangerous than seclusion; mechanical restraint is arguably more traumatic and less dignified; even manual restraints can result in fatalities. Exclusion of challenging patients may lead to their accumulation in the criminal justice system. These policy confusions and their unintended consequences may arise from failure to recognise the priority of the duty of care to safeguard patients by preventing violence by patients against patients and others. The key to the use of restrictive and intrusive interventions for preventing violence should be proportionality in their use and a demonstrable reduction in actual violence by patients against patients and others. Preventable violence by patients against patients is a failure of the duty of care for both perpetrator and victim.

The Council of Europe Committee for the Prevention of Cruel and Inhumane Treatment and Torture (CPT) has recently issued revised standards on means of restraint [15] that do not propose blanket bans on one or other form of restrictive or intrusive intervention. Instead the standards acknowledge that a range of such interventions are used in accordance with professional skill and individual circumstances, with much variation between jurisdictions, and that skilled clinicians typically employ a range of interventions such as seclusion, manual restraint, mechanical restraint and rapid tranquillisation. Such interventions are permissible if used as a last resort, proportionate, least restrictive and in accordance with law.

Research concerning the appropriate use of these interventions is difficult [16, 17]. A background ethos of non-restraint and abolition of practices such as seclusion can make scientific research difficult to justify. Randomised controlled trials require the highest standard of informed and voluntary consent. However, the most severely ill patients are often unable to give or withhold informed consent [18–22]. Furthermore, detention under mental health legislation creates a de facto duress that might negate ‘voluntary’ consent. An added complication concerns the difficulty of carrying out randomised controlled trials where the outcome would be violence to others. Observational cohort studies to evaluate services are however possible and should be seen as an ethical obligation. Observational studies are largely confined to qualitative studies of patient experience which are sometimes negative [23, 24] although perceived procedural justice, insight and coercive practices may all be independent of each other [25] and some show a positive appraisal of compulsion on recovery [26]. Observational studies may also rely on the recording of official statistics which are reductive, for example recording only the most serious and reactive interventions such as seclusion or restraint, while not recording earlier, less intrusive or restrictive interventions designed to prevent escalation.

The criticism that there is lack of evidence for the effectiveness of preventive practices may arise from lack of a theoretical framework, lack of a validated system for description and lack of a system for measurement so that scientific studies have not been possible. This may be taken as a need to translate tacit knowledge into explicit knowledge [27]. The dynamics of interactions between patients and clinicians in the context of a secure ward in a forensic hospital are best conceptualised in terms of general systems theory [28], an approach that should carefully be distinguished from approaches emphasising individual care [29], even when both conceptual frameworks are not in conflict, for example in a model of care [30].

While there is some evidence that uses of restrictive and coercive practices are not reducing [16, 31, 32], there is evidence that the systematic introduction of structured risk assessment can reduce levels of violence and aggression in psychiatric wards [33, 34] and that the use of short term risk assessment to reduce violence does so by leading to appropriate interventions [35]. There is evidence that short term risk assessment reduces coercive practices [36] and aggression [37, 38] along with other preventive measures [14, 39]. The use of short term risk assessments such as Dynamic Assessment of Situational Aggression (DASA) [40] and Brøset Violence Checklist [41] on acute and intensive care wards provide a starting point for a description of the context and decision pathways involved. This in turn offers the possibility of a theoretical basis for a system of description and measurement. Daily or more frequent measures of short term risk are well validated predictors of violence and aggression in in-patient settings. The items making up these instruments are likely to be closer to causes than risks – observable behaviours such as anger, irritability, refusal to take direction are antecedent, proximate and explanatory for subsequent violence. They are likely to be steps on causal pathways to violent acts or interactions [8, 42].

A basic theoretical understanding of the prevention, treatment and management of ward based violent behaviours should include antecedents, behaviours, interventions and consequences. If the DASA is a validated measurement of risk factors or causal antecedents at the individual level, then there is a need for a descriptive measurement system for behaviours and for interventions, translating tacit into explicit knowledge [27].

We set out to devise a method for the observational study of restrictive and intrusive practices in the prevention and management of violence in a forensic hospital, a setting of therapeutic security. We reasoned that there were no clinical, quantitative descriptions of the problem behaviours and the clinical interventions already common in such settings. We were influenced by the success of the DASA [40, 43] a short-term risk assessment incorporated into daily practice on acute and intensive care wards. In keeping with general systems theory [28], we sought to study the context and antecedents, behaviours, interventions and consequences of threatening or violent acts. We recognised that in order to determine the frequency and severity of threatening, violent or disruptive behaviour there is a need for a means of distinguishing various types of behaviour and various types of intervention. These in turn should be quantifiable in terms of seriousness or severity. In emphasising the severity of violence, we were influenced by the success of the DUNDRUM-1 triage security scale as an assessment of the need for levels of therapeutic security based largely on the seriousness of actual violence rather than the risk of violence [44, 45] as well as other types of harmful, disruptive, self-defeating and self-harming behaviour. This proportionality between the seriousness of risk and the level of care appears to be a consistent aspect of hospital care generally [1].

**Fig. 1:**
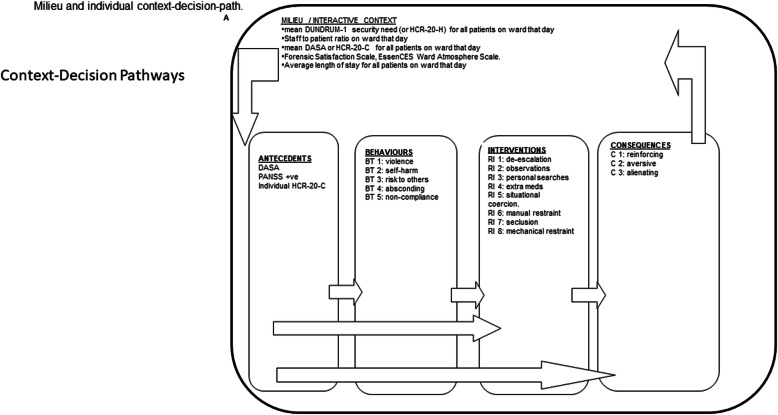
Milieu and individual context-decision-path.

Most jurisdictions have national bodies to review restrictive practice within psychiatric hospitals including secure forensic hospitals e.g. the Care Quality Commission (CQC) [13] in the UK or Mental Health Commission (MHC) [46] Ireland. Secure forensic hospitals offer care and treatment to mentally disordered offenders with a significant history of violence, often both in the community and in hospitals. Such hospitals also have a duty to provide a safe space for all patients to recover, a space where the cycle of violence is stopped and the use of violence as a tool is minimised or eliminated entirely. This is a significant challenge. While secure forensic hospitals make every effort to ensure that any restrictive practice used is necessary and proportionate to the risk in question and used for the shortest time possible, this can often be very difficult to demonstrate. The aim of the DRILL tool is to provide secure forensic hospitals with a method of reviewing the necessity and proportionality of the use of restrictive practices and testing that such practices are being used appropriately. The system described here is illustrated in Fig. 1, where context, milieu, individual and group effects can be quantified and analysed as context decision pathways consisting of antecedents (DASA on the day before), behaviours (DRILL behaviour ladders), interventions (DRILL intervention ladders) and consequences.

### Objectives

We hypothesized that (a) a minority of the patients are expected to account for a majority of incidents and interventions. (‘Pareto’ Patients). (b) forms and varieties of challenging, violent and disruptive behaviours could each be described as ordinal scales ranging from absent to the most serious; (c) these could be summated with acceptable psychometric properties including internal consistency; (d) interventions to de-escalate or where necessary, restrict and intrude on personal liberty to prevent violence could each be described as ordinal scales ranging from absent to the most serious; (e) the use of such interventions in a rational and coordinated way would be reflected by their summation with acceptable psychometric properties including internal consistency; (f) the antecedent risk measured by the DASA, the measured severity or seriousness of challenging and disruptive behaviours and the measured seriousness of interventions should correlate as a measure of proportionality; (g) measured seriousness of challenging and disruptive behaviours should correlate with measured seriousness of interventions; (h) when correcting for repeated measures, behaviour should account for interventions even when co-varying for antecedent risk (DASA score the previous day) measured as a short term assessment, medium term assessment or a measure of need for therapeutic security.

## Methods

### Study design

This is a retrospective observational cohort study for service evaluation based on case notes, incident registers and legal registers. This descriptive study is intended only as a ‘proof of concept’ study to establish some essential elements for a planned prospective study.

### Data sources

In the Republic of Ireland, the Mental Health Act 2001 requires statutory registers to be kept concerning the use of seclusion, manual restraint and mechanical restraint [46]. The authorisations, timing, nature and context of each episode thereof must be recorded on statutory forms for collation and onward reporting to the statutory regulator, the Mental Health Commission [47]. There is also a legal requirement that all medication should be recorded when prescribed and administered, with further safeguards concerning consent and documentation under the legislation. These records are subject to inspection by the Inspectorate of Mental Health Services, established by the Mental Health Act 2001 [46]. These records formed the basis for the identification of episodes of the use of restrictive and intrusive practices and the ‘context and decision pathways’ that guided them. In addition, the daily combined continuous multi-disciplinary clinical notes (including prescription and administration records for medication) provided a much more detailed and nuanced account of the context, antecedents, behaviours and consequences of such events.

### Setting

The Central Mental Hospital is the only forensic psychiatric hospital for the Republic of Ireland, serving a population of approximately 4.9 million. The hospital is divided into units (wards) according to the need for therapeutic security, ranging from acute high security to medium and low security, forming a stratified series of levels of therapeutic security along a recovery pathway from admission to pre-discharge [48–50]. All ward-based staff in the hospital are trained in de-escalation and approved methods for prevention and management of violence and aggression (PMVA).

The Central Mental Hospital Dundrum, as with all psychiatric hospitals in Ireland is subject to review by external bodies – the Mental Health Commission [46, 47], a National body, which reviews the standards of healthcare provided in all Irish mental health settings and the Committee for Prevention of Torture (CPT) of the Council of Europe. Within the hospital clinicians review the use of restrictive practices regularly as peer review. This takes place in monthly Seclusion Monitoring and Restraint Group (SMARG) meetings. This is the case internationally in most secure forensic hospitals. Clinicians and the hospital are regularly asked to justify the use of restrictive practices and to demonstrate that they were both necessary and proportionate. The aim of the development of the DRILL tool was to support the clinicians in meeting this need .

### Sample

All male patients are admitted to a 12-bed unit with a high ward-based staff-to-patient ratio (two to one in whole time equivalents). This male admission unit was built in the 1980s and modernised in 2007. Patients have single en-suite bedrooms and are locked in their rooms from 9 pm to 8 am. There are two seclusion rooms, a small timeout area, a day room and two courtyards while patients have access to a dining room at meal times and for other activities. Patients are not permitted to return to their bedrooms during the day and are expected to attend 25 h of structured activity each week, which includes psychological and occupational therapies and psycho-education [51].

Ward-based staff are male and female. The high levels of relational security reflect the recent histories of serious violence by patients in the community prior to admission and the continuing levels of risk and positive symptoms amongst the patient groups placed there [49]. Two multi-disciplinary teams, each led by a consultant forensic psychiatrist, care for the patients in the male admission unit. All nursing staff had been trained in the therapeutic management of violence and aggression. For the six-month period between 1st January 2011 and 30th June 2011 a total of 16 patients were admitted to this unit. In total, 28 patients were in scope. This paper presents our preliminary findings for patients on the male admission unit (Table 1). Relational therapeutic security levels (ward based staff to patient ratio) were consistent throughout the study period (Table 2). In this service evaluation cohort study, all eligible in-patients were included. None were excluded and none were lost to follow-up. All 28 patients studied were followed until transfer to a less acute unit, hospital discharge, or until the end of the study period.

**Table 1:** Participant characteristics

Patient variable		***n*** = 28
Mean age (years) (SD)	34.6	10.24
Gender		Male
Ethnicity	26:2	
• Irish: non-Irish	26:2	93%, 7%
Legal Status		
• Court order	4	14.3%
• Prison transfer	19	67.9%
• Not guilty by reason of insanity	2	7.1%
• Hospital transfer	3	10.7%
Diagnosis (ICD-10, SCID-I)		
• Schizophrenia	22	78.6%
• Schizoaffective disorder	3	10.7%
• Organic psychosis	1	3.6%
• Personality disorder	2	7.1%
Secondary diagnosis		
• Co-morbidity with personality disorder	14	50%
• Co-morbidity with substance misuse	25	89.3%
Index Offence		
• Homicide	4	14.3%
• Assault	8	28.6%
• Sexual Assault	0	0%
• Arson	2	7.1%
• Acquisitive Offences	4	14.3%
• Drug offences	3	10.7%
• Other	4	14.3%
• Not charged	3	10.7%
	mean	SD
Mean time at risk (days) (SD)	79.3	64.5
Mean HCR-20 score (SD)	23.4	5.7
Mean HCR-20 clinical items score (SD)	6.0	2.2
Mean DUNDRUM-1-9 item score	2.9	0.5
Mean DUNDRUM-1-11 item score	2.6	0.5
Mean forensic satisfaction survey (SD)	4	0.82
Mean EssenCES ward atmosphere score (SD)	10.5	4.92
Mean staffing ratio permanent: non permanent	5.6:2.4

**Table 2:** Actual nursing staffing levels on 12 bed ward over the course of the period of observation

Month	Mean	Mode	Range	Total Staff	Days below roster
**January**	6.09	6	2–8	189	1
**February**	5.64	5	3–8	138	2
**March**	5.13	6	3–8	159	1
**April**	7.03	5	2–8	211	0
**May**	5.19	4 and 6	3–8	161	4
**June**	4.36	4	1–7	131	3
**Total**	**5.57**	**6**	**1–8**	**989**	**11**

### Variables

#### Need for therapeutic security, risk of violence

The DUNDRUM-1 assessment [44, 45, 52–57] was used to measure need for the appropriate level of therapeutic security. The DUNDRUM-1 is a static measure completed prior to admission. An 11 item score is calculated which includes need for suicide prevention while a 9 item score includes only items relevant to violence. Each item is calibrated in units of meaningful change from 0 to 4, so that an item scored 0 indicates no need for admission, 1 indicates a need for an open ward placement, 2 indicates low security, 3 medium security and 4 high security. A mean item score is calculated by adding all items and dividing by the number of items. A mean DUNDRUM-1 item score of 3 to 4 indicates need for high security, 2 to 3 indicates medium security, 1 to 2 indicates low security and a lower mean item score is in keeping with admission to an open ward. Cronbach’s alpha for internal consistency in this sample was 0.668, reflecting the relatively narrow range of scores for patients admitted.

The Historical, Clinical, Risk Management-20 (HCR-20) [58] is completed soon after admission and at six monthly intervals. Data were taken from those assessments completed prior to the study period for those already in hospital at the commencement date and within two weeks of admission for those admitted during the study period. Cronbach’s alpha for the HCR-20-C items – a measure of current dynamic risk of violence – was 0.642.

The mean scores for these instruments may be taken as a measure of the ‘ambient’ risk and seriousness of the risk over the medium term. Similarly, ward atmosphere was measured using the EssenCES [59]. The Forensic Satisfaction Scale (FSS) [60] and Suicide Risk Assessment and Management Manual (S-RAMM) [61, 62] were collected but completed by too few patients to be analysed in this part of the study.

### Antecedent factors - milieu

The DASA [40] is an assessment tool primarily based on observation. It was completed daily by nursing staff. Seven items are rated absent or present and include: impulsivity, unwillingness to follow directions, irritability, sensitivity to perceived provocation, easily angered when requests denied, negative attitudes and verbal threats. The DASA score allows an individual management of risk as well as cumulatively providing a contextual assessment of the ward environment. The mean DASA score on any given day may be taken as a measure of the ambient risk or level of disturbance on the ward on a given day. The patient’s primary nurse routinely calculated this each day at 18.00 h. Cronbach’s alpha was 0.942. Reliable Change Index (RCI) [63] was 0.56 (range 0–7) indicating that for an individual patient, a unit change was reliable. In an explanatory system consisting of fixed risk factors, stable dynamic risk factors, acute dynamic risk factors and triggers, these imminent items are acute dynamic risk factors [64, 65] though they may also be regarded as causal.

We used the DASA for the previous day as an independent predictor and as a co-variate in models of behaviour and interventions on the following day.

### Staffing as context

The use of non-permanent staff is recognised to lead to inconsistency and a higher rate of violent incidents [66, 67]. We recorded the ratio of actual staff to patients for each day, using only those staff allocated to work consistently on that particular unit for the study period. Staff brought in from other units on a casual basis to fill gaps in the rotas were categorised as non-regular staff. The service does not use agency staff. The hospital did not use unqualified nursing staff at the time of this study. These data will be described in a subsequent paper.

### Dundrum restriction intrusion liberty ladders (DRILL)

#### Development process

This framework was derived following a preliminary review of the literature and clinical practice. A series of rating ‘ladders’ was then drafted by HGK using a conceptual mapping process (Fig. 1). We used a modified iterative Delphi process facilitated by HGK to identify and define the elements and magnitudes of restrictive and intrusive interventions. A first draft was circulated amongst experienced forensic psychiatrists with an interest in intensive care (DM, PO’C), nurses with a wide range of experience were consulted, including accredited trainers in PMVA (DT, JT, PMcK, PB) and allied health professionals and each draft was discussed and critically reviewed amongst clinicians drawn from psychiatry, mental health nursing, social work, occupational therapy and clinical psychology. The fourth draft was considered ready for application in this preliminary study. Statements were developed to describe each adverse incident and interventions that were in turn grouped in a hierarchical manner to construct a framework of ‘ladders’. This process was repeated and refined iteratively by experienced clinicians from medicine, nursing, psychology, social work and occupational therapy.

#### Context decision pathways

Context, antecedents, behaviour, interventions, consequences were conceptualised as a series of events organised in temporal sequence - a context decision path - so that causes, interactions and effects can be considered (Fig. 1).

The Delphi process generated a taxonomy of behaviours, interventions and consequences. In the interests of dimensional congruence each ‘ladder’ was rated from 0 to 5. Each ladder was stratified into five main gradations tethered to definitions with sub-divisions for descriptive options.

For behaviours, these could broadly be summarised as: no behaviour, threats, minimal acts, moderate, serious or severe acts. Each gradation was however tethered to clear operational definitions (Table 3).

**Table 3:** DRILL Behaviour ladders

DRILL Behaviour ladders				***n*** = 2175	(%)	
**Violence**				88	(4)	
**Self-harm**				99	(4.5)	
**Placing others at risk**				246	(11.4)	
**Escape**				42	(1.9)	
**Non-compliance with treatment**				154	(7.1)	
**Total**				**629**	**(28.9)**	
**Rating**		**Violence**	**Self-harm**	**Placing others at risk**	**Escape**	**Non-compliance with treatment**
**No behaviour**	**0**	2062	2054	1907	2111	1999
**Threats**	1	78	85	190	32	49
**Minimal act**	2	9	14	50	1	76
**Moderate**	3	1	0	6	9	13
**Serious**	4	0	0	0	0	2
**Severe**	5	0	0	0	0	14
**Missing data**		25	22	22	22	22
**Total**		2175	2175	2175	2175	2175

For interventions, these gradations corresponded to: no intervention, anticipation, reaction, intrusion, restriction, constraint where zero represents no obvious restriction or intrusion over the ordinary status of in-patient and five represents the most serious intervention legally permitted (Table 4).

**Table 4:** DRILL Intervention Ladders: Contingency management interactions

DRILL Intervention Ladders:						***n*** = 2175	(%)		
De-escalation						188	(8.8)		
Restriction of privacy						401	(18.4)		
Intrusion of bodily integrity (medication)						101	(4.6)		
Physical restraint						85	(3.9)		
Restriction on the use of space						380	(17.6)		
Restriction of bodily space & intrusion of bodily integrity						146	(6.7)		
Mechanical restraint						0	(0)		
Situational coercion						2093	(96.2)		
**Total**						**3394**			
**Rating**		**De-escalation**	**Observations / restriction of privacy**	**Personal searches**	**Extra medication**	**Situational Coercion**	**Manual restraint**		**Restraint (mech’l)**
**No intervention**	0	1965	1770	2013	2058	0	2074	1779	2159
**Anticipation**	1	105	320	0	65	1994	66	79	0
**Reaction**	2	23	28	1	14	4	5	229	0
**Intrusion**	3	8	0	80	21	4	5	0	0
**Restriction**	4	51	37	65	1	44	9	14	0
**Constraint**	5	1	16	0	0	47	0	58	0
**Missing data**		22	4	16	16	82	16	16	16
**Total**		2175	2175	2175	2175	2175	2175	2175	2175

Each behavioural ladder and each intervention ladder should be rated for any single incident dependent on the circumstances, severity and the duration of the context decision path. In a day, multiple episodes can be rated and then summated, or the highest scoring episode can be rated for the day. In this study, the highest scoring episode was taken.

### DRILL behaviour ladders

The behaviour ladders (DRILL-B) are summated because we conceptualised all of these behaviours as being on a continuity with and additive with other threatening or harmful behaviours such as actual violence, or self-harm. In order to prevent imminent violence, skilled clinicians continuously assessed all of these behaviours and used clinical judgement to assess the imminence and seriousness of threatened harm or actual violence. This is done in a planned, synchronised and progressive way, guided also by the DASA ratings in order to anticipate and to plan proportionate interventions, based on a knowledge of the individual patient’s risk assessment, history of violence, mental state, vulnerabilities and advance preferences.

### DRILL behaviour, intervention and consequences ladders

Behaviours identified included violence, self-harm, risk to others, absconding (escaping) and non-compliance with treatment. Each of these behaviours was considered to hold the possibility of leading to restrictive interventions. Each of these adverse incidents equated to a single incident for that type of behaviour. However, the DRILL Behaviour ladders can also occur together in a context decision pathway and may correlate with each other. For example a patient may be non-compliant with medication and may then attack staff trying to administer medication. Such an action - while a single incident - would be recorded under both headings above.

DRILL Interventions (DRILL-Int) included de-escalation, observations, personal searches, extra medication, situational coercion, manual restraint, seclusion and mechanical restraint. De-escalation was considered to encapsulate a range of interventions from normal communication to conflict resolution. Special nursing observations are used extensively both in the UK [68] Ireland and internationally. This procedure resulted in a restriction of privacy and an intrusion on the patient’s personal space. Likewise, the use of extra medication above and beyond the prescribed regular daily treatment would compromise the patient’s bodily integrity either by the route of administration, the extra dosage or the level of force used. Forensic mental health also includes a number of security-orientated tasks such as personal searches and room searches. While consensual when passing through airports, the loss of dignity and loss of implied consent in hospital, along with removal of possessions or the removal of day clothes constituted a restrictive, intrusive or coercive practice.

In Ireland, seclusion is defined as the supervised confinement of the person alone in a room where staff observe continuously from the outside. Seclusion is the most restrictive of the five gradations on restrictions of the use of space [47]. Physical or manual restraint refers to the immobilisation of the person by two or more staff (i.e., manual control and restraint). In this study, mechanical restraint did not extend to the use of straightjackets, Pinel restraints or similar devices, practices or equipment since these were never used. A rating for mechanical restraint was included to allow for international comparisons. Manual restraint, seclusion and mechanical restraint can only be initiated in Ireland by a registered mental health practitioner and must be authorised in accordance with law by a medical practitioner and consultant psychiatrist at fixed, frequent intervals with a view to early termination.

A last set of interventions defined a set of sanctions that are considered legitimate in providing a safe environment: detention under mental health legislation, the use of behavioural treatment programmes and the presence of more than one member of staff during negotiations.

The DRILL intervention ladders are summated because all of these practices were conceptualised as being on a continuity with and additive with legally regulated practices such as seclusion or restraint. In order to prevent imminent violence, skilled clinicians use anticipation and clinical judgement to deploy not one but a range of the interventions described and quantified here. This is done in a planned, synchronised and progressive way, proportionate to the seriousness and imminence of the threat [14, 35, 39]. The vulnerabilities and advance preferences of the individual can also be taken into account. In this way not only can violence and harm be prevented, but the use of the most intrusive and restrictive interventions such as seclusion and restraint can be minimised.

The context decision path ends with three ladders describing the consequences of the behaviour and interventions and is intended to help structure a ‘debriefing’. In the spirit of an interactive intervention, the debriefing should seek to explore the subjective experiences of the patient, both reinforcing and aversive, as well as the staff perceptions of alienation if any. This stage can be seen as completing the analysis of a context decision path, by exploring the various facets of the events from varying points of view.

### DRILL toolkit

This series of rating ‘ladders’ was structured into an audit toolkit that allowed a qualitatively derived quantitative analysis of the use of restrictive and intrusive interventions. This toolkit (DRILL: Dundrum Restrictive-Intrusion of Liberties Ladders) was structured into a handbook and is available on www.tara.tcd.ie [69].

### Context decision path

A context decision path (CDP) organises the ladders into a chronological sequence (Fig. 1). This allowed analysis of any series of events and associated interventions over a meaningful period of time such as a nursing shift, a 24 h cycle of shifts or for the duration of an adverse episode and related interventions such as seclusion. Each context decision path commences with the antecedent risk (DASA) for the day in question or the previous day and includes the ratings for subsequent behaviours and interventions. Each ends when the last intervention is concluded. For the purposes of this study, each set of observations ends each 24 h.

In so far as possible, the daily assessment of risk using the DASA tool, or similar instrument, should define the starting point for a context decision path. This allows clinicians to avoid the uninformative formulation of ‘unprovoked incident’, concentrating instead on the analysis of antecedent context and mental state, the behaviour itself and the consequences for all concerned, whether reinforcing, alienating or neutral.

### Data sources and measurement

The registers and records for each patient who spent any time on any of these units were systematically collated and used to rate the outcome measures. All data were collected by two experienced clinicians, entered first into Excel and then in SPSS-25 [70] for analysis. The recording of adverse incidents was elicited from the hospitals’ statutory clinical practice forms for seclusion, mechanical restraint and physical restraint. These incidents were cross-referenced to the hospital’s incident forms for health and safety. The incidents were also cross-referenced with a review of the daily entries in the multidisciplinary clinical notes, medication prescription charts and daily reports to nursing administration for all 181 days. These data were finally cross-referenced with data submitted to the Mental Health Commission and with the register of admissions and discharges for that period.

Two anonymised SPSS databases were collated. One Excel database was established to record daily DASA scores by patient, generating a line of data for each day for each patient present on the unit that day (181 X 12 = 2172 patient-days). A second database recorded the mean measures for all patients present for each day, generating 181 lines of data, one for each day. The second data base will be analysed for milieu / contextual effects in a subsequent paper.

### Statistics

All calculations were carried out using SPSS-25 [70]. Cronbach’s alpha statistic was used to assess internal consistency [61]. The Reliable Change Index (RCI) [63] was calculated for the mean item score for DRILL-Behaviour (sum of five items divided by five to yield a range from 0 to 5) and for mean item score for DRILL-Interventions (sum of seven items excluding mechanical restraint, divided by seven to yield a range from 0 to 5) using Cronbach’s alpha [63, 71] as a guide to the reliability of unit changes in each measure. If the RCI is less than one unit of change, then at the individual level, a unit of change is significant.

Inter-rater reliability could not be assessed in a meaningful way by rating the same material twice. The internal consistency of the newly developed scales was calculated using Cronbach’s alpha statistic.

Spearman’s rank correlation test was used for preliminary exploratory tests of hypotheses.

The receiver operating characteristic area under the curve (ROC-AUC) was not used as a measure of predictive power when antecedent events were compared with subsequent events because of the repeated measures on the same patients inherent in this design and this clinical context.

In a model in which there is extensive (daily) repeated measures on the same subjects, we opted not to use data smoothing or seasonal adjustments [72] because the time period of six months and limited number of subjects involved made seasonal adjustment potentially misleading. Similarly the exclusion of outliers would be likely to lose important data since it is normal for a small proportion of patients to account for the majority of incidents. Since this was not an A-B study of a change in practice, a corrected regression analysis [35] was also not appropriate. Recent progress in correcting for repeated measures in similar settings and paradigms to this [36–38] indicated the use of General Estimating Equations (GEE). Repeated measures in the same individuals were studied using General Estimating Equations in SPSS-25 [70]. A custom model was used in all cases, with normal distribution and identity as the link function. Case number was used to identify the subject variable, day number (1 to 181) as within-subject variable. A main effects model was used for model building using the independent factor then adding covariates, with intercept not included in the model. The scale parameter estimate was maximum likelihood estimation. Model effects analysis was type III and 95% confident intervals. Wald X^2^ statistics were calculated. Corrected Quasi Likelihood under Independence Model Criterion (QICC) was used to test goodness of fit, with information criteria in ‘smaller-is-better’ form. Competing models were compared on this basis. Marginal means for the independent determinant were calculated.

In a preliminary scoping exercise using 181 days of data for a six bed male intensive care unit, various levels of autoregression were compared with an independent structure for the working correlation matrix. In each model, ‘independence’ yielded a lower QICC than any level of autoregression.

### Study size

A goal of this study was to allow calculation of effect sizes that would inform the calculation of power for future prospective studies. The data accumulating for 28 patients in 12 beds over 181 days allowed tests of correlation. Correcting for repeated daily measures within individuals (mean days at risk 6.46) also allowed calculation of effect sizes.

### Qualitative variables

Demographic details were recorded for diagnosis, age, length of stay and index offence (Table 1). All patients were detained under mental health legislation. Other variables included co-morbidity with either personality disorder or substance misuse as neither of these diagnoses makes up part of the legal criteria for detention under Irish mental health legislation. A wealth of qualitative material was accumulated from the Delphi process and this is summarised informally.

### Staffing levels

The standard staffing levels for this ward during the period studied were eight ward-based nurses per day and three ward- based nurses at night and the team is expected to absorb the first special observation within these numbers. Nursing staff work 13-h and 11- h shifts on a ‘two days on, two days’ off roster. Of the 57 episodes of special observations at 1:1 nursing, 39 required additional staff. Table 2 shows the actual staffing levels over the course of the study period. The hospital utilises overtime from within the service to manage gaps in the roster but 66% of the nursing team were regularly allocated to the unit for that period with a mean of 5.57 regular staff.

## Results

### Participants

The unit had at least 100% bed occupancy for the study period equating to 12 beds for 181 days. This accounted for 2172 bed days or context decision paths (CDP) where one CDP equates to one 24-h bed day. It exceeded 100% bed occupancy on 3 occasions.

The clinical and legal characteristics are summarised in Table 1. Most patients had schizophrenia (79%) or schizoaffective disorder (11%) with co-morbid substance misuse and personality disorder recorded for 50% and 89.3% of the patients respectively. Violent offences were the most common reason for being in custody (43%) while 11% had not been charged with an offence. The majority (67.9%) were admitted on transfer from prison. Ward atmosphere (EssenCES) was comparable to other studies.

The number of days ‘at risk’ during the 181 day study period for the 28 patients in scope was mean 79.3 (SD 64.5), median 55, range 2 to 180.

The DUNDRUM-1 mean item score (DUNDRUM-1 nine item 2.9, SD 0.9, range 2.4–3.9; DUNDRUM-1 11 item 2.6, SD 0.5) was in keeping with a medium secure population of patients with some scoring in the high secure range.

Hypothesis a: a minority of the patients are expected to account for a majority of incidents and interventions (Pareto Patients).

There were 16 admissions to the unit during the period of observation. Two of the 28 patients accounted for 53% of seclusion episodes but were amongst the 12 who were admitted prior to the observation period. Five (18%) of the 28 patients in scope accounted for approximately 80% of all incidents including 77% of all seclusions.

Hypothesis b: forms and varieties of challenging, violent and disruptive behaviours could each be described as ordinal scales ranging from absent to the most serious.

Assessment scales were composed for common adverse behaviours (Five ladders: violence, self-harm, risk to others, absconding and non-compliance with treatment.

Hypothesis c: these could be summated with acceptable psychometric properties including internal consistency.

The five DRILL Behaviour ladders (items) had Cronbach’s alpha = 0.720, increasing to 0.740 when self-harm is omitted. For dimensional congruence, the scores for the five ladders were summated and divided by five to yield a mean score with range 0 to 5. The Reliable Change Index for the mean score was 0.36.

There were 629 recorded incidents over the period of 181 days that could be rated anywhere on any of the DRILL Behaviour ladders (Table 3). We encountered no episodes above level 3 in any of the DRILL Behaviour ladders with the exception of ‘non-compliance with treatment’, where 16 incidents were recorded at a serious or severe level, scoring 4. or 5, describing a complete refusal to accept or engage in treatment leading to a risk of violent conduct.

Hypothesis d: interventions to de-escalate or where necessary, restrict and intrude on personal liberty to prevent violence could each be described as ordinal scales ranging from absent to the most serious.

A set of interventions was formed by the same process into eight ‘ladders’: de-escalation, observations, personal searches, extra medication, situational coercion, manual restraint, seclusion, mechanical restraint (Table 4). Each ladder was again composed of verbal definitions of ordinal ratings and for dimensional congruence each was scored from zero to five.

Hypothesis e: the use of such interventions in a rational and coordinated way would be reflected by their summation with acceptable psychometric properties including internal consistency.

Cronbach’s alpha calculated for seven DRILL Intervention ladders (items, omitting mechanical restraint which was not used) = 0.876, and for the mean of the summated item scores (range 0 to 5), Reliable Change Index = 0.50.

Table 4 shows that there were 3394 interventions that could be scored anywhere on the DRILL Interventions ladders. The most frequent was situational coercion which was rated positive for 2093 incidents, every patient every day. However this arises from 1994 patient-day-occasions when this was rated ‘1’, the lowest level, in keeping with the locked and intensively staffed nature of the unit. Concerning de-escalation, there was only a single incident when a patient was moved to another unit; there were only 16 incidents when a patient was observed at two to one level within arm’s length – the same patient on more than one series of days; personal searches never extended to body cavity searches; extra medication never extended to electro-convulsive therapy and only once extended to intramuscular injection in a crisis, with 21 incidents of initiating rapid acting long acting anti-psychotic medication by injection; situational coercion extended to a three person team deployed during a negotiation e.g. regarding medication on 47 occasions; manual restraint never progressed to a prone position but involved degrees of restraint from pain-free immobilisation of wrists, arms and control of the head through to controlled descent to a supine position on 9 occasions; the restriction of use of space extended to seclusion as legally defined in 58 episodes, although there were many lesser interventions involving restricted use of space; mechanical restraints were not used in any form or degree.

### Correlations

Neither DUNDRUM-1 nor DUNDRUM-2 correlated with adverse incidents (DRILL B) or intervention ladders (DRILL Int.) with the exception of ‘observation levels’ where DUNDRUM-1 correlated, Spearman’s r = 0.523 and DUNDRUM-2 r = 0.544.

Neither legal status nor the nature of the index offence showed any positive relationship with adverse incidents or restrictive interventions.

There was no correlation between HCR-20 or any of its sub-scales and the DRILL Behaviour or DRILL intervention ladders.

Hypothesis f: antecedent risk should correlate with measured seriousness of behaviours and measured seriousness of interventions.

Table 5 shows that DASA score on the previous day had a statistically significant relationship with all of the DRILL behaviour ladders except for self-harm. DASA for the previous day also correlated with each of the DRILL intervention ladders except for mechanical restraint as expected because there were no episodes of mechanical restraint. DASA for the previous day correlated higher with observation levels (‘restriction of privacy’ r = 0.392) and situational coercion (0.358), which reflects the strategy of increasing nursing levels in the first instance prior to more coercive measures such as physical restraint (0.213) or seclusion (‘restriction on use of space’ r = 0.277).

**Table 5:** DASA on previous day, DRILL Behaviours and DRILL Interventions. Spearman rank correlation coefficients

DRILL Behaviour ladders	DASA Previous Day	DASA Same Day
• Violence	0.168	0.304
• Self-harm	0.004	0.011
• Placing others at risk	0.259	0.317
• Escape	0.135	0.123
• Non-compliance with treatment	0.400	0.455
**DRILL Interventions ladders**		
• De-escalation	0.314	0.375
• Restriction of privacy	0.392	0.414
• Medication	0.275	0.362
• Physical restraint	0.213	0.317
• Restriction on use of space	0.277	0.308
• Restriction on bodily space & integrity	0.328	0.411
• Mechanical restraint	0	0
• Situational coercion	0.358	0.431

Table 6 shows that for 2166 patient-days, DASA measured on the previous day correlated significantly with all DRILL Behaviour ladders and summated total DRILL Behaviour score except self-harm, though effect sizes were small (r = 0.106 to 0.261) except for a moderate association with non-compliance (r = 0.380). Similarly DASA the previous day correlated with all DRILL intervention ladders and the summated total, with moderate or large associations (r = 0.334 to 0.703) except for a small association with manual restraint (r = 0.267).

**Table 6:** Drill Behaviour Ladders and DRILL Intervention Ladders and Scales: Context decision pathways

	Violence	Self-harm	Risk to others	Escape	Non compliance	DRILL Behaviour scale (total)	DASA previous day
**De-escalation**	0.691	0.386	0.745	0.401	0.653	0.734	0.309
**Observation**	0.206	0.236	0.507	0.264	0.421	0.595	0.398
**Searches**	0.415	0.078	0.440	0.394	0.600	0.452	0.314
**Extra meds**	0.375	0.114	0.319	0.172	0.581	0.334	0.256
**Situational coercion**	0.427	0.172	0.529	0.528	0.764	0.544	0.356
**Manual restraint**	0.400	0.026	0.266	0.072	0.414	0.267	0.186
**Seclusion**	0.459	0.456	0.470	0.265	0.452	0.570	0.270
**Mechanical restraint**	–	–	–	–	–	–	
**DRILL Intervention scale (total)**	0.409	0.394	0.632	0.253	0.424	0.703	0.351
**DASA previous day**	0.160	0.000	0.261	0.138	0.380	0.252	

Spearman rank correlation coefficients. For DRILL ladders *n* = 2174, for DASA previous day *n* = 2166. All p < 0.001 except for some self-harm correlations

Hypothesis g: measured seriousness of challenging and disruptive behaviours should correlate with measured seriousness of interventions.

Table 6 also shows that for 2174 patient-days (context decision pathways) all DRILL Behaviour ladders and all DRILL intervention ladders, and the total scores, all correlated significantly except for self-harm and manual restraint. Effect sizes were small for the associations of self-harm with most types of intervention. Each DRILL behaviour ladder had a medium or large association with the DRILL intervention summated scale, while each DRILL intervention ladder had a moderate or large association with the DRILL Behaviour ladders except for manual restraint, where associations were understandably small for self-harm, risk to others and escape behaviours.

Hypothesis h: when correcting for repeated measures, behaviour should account for interventions even when co-varying for antecedent risk (DASA score the previous day) measured as a short term assessment, medium term assessment or a measure of need for therapeutic security.

### General estimating equations (GEE) for all patient-days

#### Model 1

Table 7 shows the effect of DASA score for the previous day on DRILL-Behaviour scores. The antecedent DASA score had a significant effect on the DRILL-Behaviour score the following day (Wald X^2^ = 146.7, df = 8, *p* < 0.001). Including the DUNDRUM-1 nine item score for seriousness of past violence and need for therapeutic security as co-variate did not demonstrate any effect of DUNDRUM-1 score; similarly including the HCR-20-C score for current dynamic risk of violence demonstrated no effect of this measure of dynamic risk, nor did a model in which both DUNDRUM-1 and HCR-20-C were included as co-variates.

**Table 7:** General Estimating Equations Model 1: DASA previous day as independent, DRILL-Behaviour as dependent, HCR-20-Current scale (HCR-20-C) and DUNDRUM-1 (D-1) as co-variants

model	independent	Co-variant	dependent	n	n	QICC	Parameter	Wald X^**2**^	df	p
1.0	DASA previous day	–	DRILL-B	2166	28	2368.7	DASA previous day	146.7	8	0.000
1.1	“		“	“	“	2354.3	DASA previous day	211.1	8	0.000
		D-1					D-1	0.656	1	0.416
1.2	DASA previous day		DRILL-B	2103	25	2341.7	DASA previous day	121.5	8	0.000
		HCR-20-C					HCR-20-C	0.304	1	0.582
1.3	DASA previous day		DRILL-B	2103	25	2321.5	DASA previous day	237.6	8	0.000
		D-1	DRILL-B				D-1	0.797	1	0.378
		HCR-20-C					HCR-20-C	0.011	1	0.915

#### Model 2

Table 8 shows the effect of DASA score for the previous day on DRILL-Intervention scores. The antecedent DASA score had a significant effect on DRILL-Intervention score the following day (Wald X^2^ = 1034.0, df = 8, *p* < 0.001). Including the DUNDRUM-1 nine item score for seriousness of past violence and need for therapeutic security as co-variate did not demonstrate any effect of DUNDRUM-1 score; similarly including the HCR-C score for current dynamic risk of violence demonstrated no effect of this measure of dynamic risk, nor did a model in which both DUNDRUM-1 and HCR-20-C were included as co-variates.

**Table 8:** General Estimating Equations Model 2: DASA previous day as independent, DRILL-Interventions total (DRILL-Int.) as dependent. DUNDRUM-1 (D-1) and HCR-20-Current scale (HCR-20-C) as co-variants

model	independent	Co-variant	dependent	n	N	QICC	Parameter	Wald X^**2**^	df	p
2.0	DASA previous day	–	DRILL-Int	2166	28	17,991.0	DASA previous day	1034.0	8	0.000
2.1	DASA previous day		DRILL-Int	2166	28	17,771.9	DASA previous day	829.9	8	0.000
		D-1					D-1	2.5	1	0.113
2.2	DASA previous day		DRILL-Int	2103	26	17,116.4	DASA previous day	919.8	8	0.000
		HCR-C					HCR-C	2.4	1	0.121
2.3	DASA previous day		DRILL-Int	2103	26	16,978.3	DASA previous day	1029.8	8	0.000
		D-1					D-1	1.745	1	0.187
		HCR-C					HCR-C	2.86	1	0.091

#### Model 3

Table 9 shows the effect of DRILL-Behaviour score on DRILL-Intervention score. The DRILL-Behaviour score had a significant effect on DRILL-Intervention score (Wald X^2^ = 262,158.6, df = 9, *p* < 0.001). Including the DASA score for the previous day as co-variate did have a significant effect (Wald X^2^ = 14.3, df = 1, *p* < 0.001) though the effect of DRILL-Behaviour score on DRILL-Intervention score remained significant (Wald X^2^ = 40,737.0, df = 9, *p* < 0.001). Including the DUNDRUM-1 nine item score for seriousness of past violence and need for therapeutic security as co-variate did not demonstrate any effect on the relationship between DRILL-Behaviour score and DRILL-Intervention score. Similarly including the HCR-20-C score for current dynamic risk of violence demonstrated no effect of this measure of dynamic risk on the relationship between DRILL-Behaviour score and DRILL-Intervention score, nor did a model in which both DUNDRUM-1 and HCR-20-C were included as co-variates. An omnibus model with DRILL-Behaviour score as independent variable, DRILL-Intervention score as dependent variable and DUNDRUM-1, HCR-20-C and DASA previous day as co-variates again showed a significant effect of DRILL-Behaviour score on DRILL-Intervention score (Wald X^2^ = 154,977, df = 11, *p* < 0.001) with only DASA score for the previous day having a significant co-variant effect (Wald X^2^ = 11.5, df = 1, *p* = 0.001).

**Table 9:** General Estimating Equations Model 3: DRILL-Behaviour total (DRILL-B) as independent, DRILL-Interventions (DRILL-Int.) as dependent. DASA previous day, DUNDRUM-1 (D-1) as co-variants

model	independent	Co-variants	dependent	n	N	QICC	Parameter	Wald X^**2**^	df	P
3.0	DRILL-B	–	DRILL-Int	2177	28	8233.2	DRILL-B	262,158.6	9	0.000
3.1	DRILL-B		DRILL-Int	2166	28	6437.9	DRILL-B	40,737.0	9	0.000
		DASA previous day					DASA previous day	14.3	1	0.000
3.2.1	DRILL-B		DRILL-Int	2177	28	8163.1	DRILL-B	132,781.3	10	0.000
		D-1					D-1	2.09	1	0.148
3.2.2	DRILL-B		DRILL-Int	2166	28	6365.1	DRILL-B	182,993.0	10	0.000
		D-1					D-1	2.4	1	0.121
		DASA previous day					DASA previous day	13.7	1	0.000
3.3.1	DRILL-B		DRILL-Int	2144	25	7075.1	DRILL-B	97,063.1	10	0.000
		HCR-20-C					HCR-20-C	3.6	1	0.058
3.3.2	DRILL-B		DRILL-Int	2103	25	5355.5	DRILL-B	127,772.7	10	0.000
		HCR-20-C					HCR-20-C	3.28	1	0.070
		DASA previous day					DASA previous day	11.59	1	0.001
3.33	DRILL-B		DRILL-Int	2144	25	7073.4	DRILL-B	124,367	11	0.000
		HCR-20-C					HCR-20-C	3.68	1	0.055
		D-1					D-1	0.18	1	0.671
3.34	DRILL-B			2103	25	5351	DRILL-B	154,977	11	0.000
		HCR-20-C					HCR-20-C	3.41	1	0.065
		D-1					D-1	0.416	1	0.519
		DASA previous day					DASA previous day	11.5	1	0.001

### Validation by item analysis

Using GEE, Tables in Supplemental materials show the effect of DASA score for the previous day on DRILL behavioural total score and on DRILL violent behaviour score (p < 0.001), self-harming behaviour score (NS), placing others at risk score (p < 0.001), escape behaviour score (p < 0.001), non-compliance score (p < 0.001). The effect of DASA score for the previous day on DRILL interventions total score was significant (p < 0.001), as was DASA previous day on de-escalation score (p < 0.001), DRILL observations score (p < 0.001), DRILL extra medications score (p < 0.001), DRILL physical (manual) restraint score (p < 0.001), DRILL seclusion score (p < 0.001), DRILL searches score (p < 0.001) and DRILL situational coercion score (p < 0.001). Also using GEE, the effect of DRILL Behaviours total score on DRILL interventions total score was significant (p < 0.001) as was the effect of DRILL behaviours total score on DRILL de-escalation score (p < 0.001), DRILL observations score (p < 0.001), DRILL extra medication score (p < 0.001), DRILL physical (manual) restraint score (p < 0.001), DRILL seclusion score (p < 0.001), DRILL searches score (p < 0.001), and DRILL situational coercion score (p < 0.001).

## Discussion

We found that the DRILL tool had good psychometric properties and was a reliable method of rating both challenging, violent and disruptive behaviour as well as the subsequent staff-led response. We found that the DRILL tool was useful as a real-world method (Fig. 1) for testing the proportionality of restrictive practices used in a secure forensic hospital for a range of common interventions ranging from increasing observational levels, to seclusion, forced medication and restraint.

We have shown that (a) even amongst psychiatric patients in a secure forensic hospital, selected because of their need for treatment in conditions of therapeutic safety and security, a minority of patients accounted for the majority of problem behaviours and the majority of interventions [7], characterised here as ‘Pareto’ patients; we observed that the majority of incidents – closely approximating to 80% were accounted for by approximately 20% of the patients, as were the majority of interventions, including seclusion. This ‘Pareto’ [7] phenomenon was described as a generalisation of an economic principle and is widely used in quality control [73]. This observation, which is not new, confirms the utility of the model of care for forensic hospitals described as stratified therapeutic security [1, 48, 49].

(b) forms and varieties of challenging, violent and disruptive behaviours could each be described as ordinal scales ranging from absent to the most serious. A Delphi process generated five ordinal scales for challenging and disruptive in-patient behaviours including violence, self-harm, risk to others, absconding and non-compliance. The same process generated eight ordinal scales for restrictive and intrusive interventions to prevent violence including de-escalation, observations, personal searches, extra medication, situational coercion, manual restraint, seclusion and mechanical restraint. Three ordinal scales for consequences - reinforcing, aversive and alienating, have not been examined further in this preliminary study [69, 74].

The DASA items also had good internal consistency and reliability as indicated by Cronbach’s alpha = 0.924, with all items contributing positively, yielding a Reliable Change Index = 0.56 indicating that a unit change in this score was reliable at the individual level.

We have shown that (c) the DRILL Behaviour ladders (items) could be summated with acceptable psychometric properties including internal consistency. The five DRILL Behaviour ladders (scales) summated with Cronbach’s alpha = 0.720, indicating acceptable internal consistency or reliability. The Reliable Change Index for the mean item score (total divided by number of items) was 0.36, indicating that a unit change in an individual was reliable.

We have shown that (d) interventions to de-escalate or where necessary, restrict and intrude on personal liberty to prevent violence could each be described as ordinal scales ranging from absent to the most serious.

We have shown that (e) the DRILL Interventions appeared to be used in a rational and coordinated way as reflected by their summation with acceptable psychometric properties including internal consistency. Seven of the eight Intervention ladders (omitting mechanical restraint) summated with Cronbach’s alpha = 0.876, Reliable Change Index = 0.50, indicating that a unit change in the total score was reliable.

We explored the extent to which (f) the antecedent risk measured by the DASA, the measured severity or seriousness of challenging and disruptive behaviours and the measured seriousness of interventions correlated as a measure of clinical proportionality. Table 5 shows these correlations. DASA score on the previous day had weaker correlations with each item of the DRILL Behaviour ladders than did the DASA on the same day, with ‘self-harm’ consistently least correlated and ‘non-compliance with treatment’ consistently best.

We tested whether (g) measured seriousness of challenging and disruptive behaviours correlated with measured seriousness of interventions. Table 6 shows that this held true, item by item for each of the five DRILL Behaviour ladders and each of the DRILL Intervention ladders, excluding mechanical restraint. The summated DRILL Behaviour scale (total score) also correlated with each of the DRILL Intervention ladders (items) and the summated DRILL Intervention ladders (total score) correlated with each of the DRILL Behaviour ladders (items). This strongly indicates that staff used restrictive, intrusive and coercive practices in a way that was proportionate to disruptive and challenging, violent behaviour. Of note, the DASA score for the previous day also correlated with each item of the DRILL Behaviour ladders and each item of the DRILL interventions, except for self-harm, indicating a possible confounding effect. This was addressed in a series of General Estimating Equations models.

We examined whether, (h) when correcting for repeated measures, summated DRILL Behaviour scores accounted for summated DRILL Interventions even when co-varying for antecedent short term risk (DASA score the previous day), medium term risk (HCR-20-C) or a measure of need for therapeutic security (DUNDRUM-1). Using General Estimating Equations to correct for repeated measures, we found that DASA score on the day before remained as a significant predictor of both DRILL Behaviours and DRILL Interventions, total scores and individual items. GEE also showed that DRILL Behaviours accounted for significant variance in DRILL Interventions, and this remained true even though DASA from the previous day was a significant co-variant.

We have established a taxonomic descriptive system (Fig. 1) in which a range of challenging and harmful behaviours can each be defined and quantified on ordinal scales of severity or seriousness (DRILL-Behaviours). These can be added to generate a scale that has internal consistency and reliability. A range of restrictive, intrusive and coercive interventions has also been defined and quantified on ordinal scales of severity or seriousness, and these can also be added to generate a scale with internal consistency and reliability (DRILL-Interventions). Measured behaviours correlate item by item with measured interventions and when correcting for repeated measures, total scores of behaviours and interventions are significantly related even when covarying for short term antecedent risk (DASA) and other measures of risk (HCR-20) and need for therapeutic security (DUNDRUM-1).

### Limitations

This is an atheoretical approach to the biological, psychological, social, institutional or individual causes of violence and the means of preventing violence [75] in psychiatric hospitals. We have sought instead to provide a framework (Fig. 1) for further scientific research [27]. Serious or severe acts of violence are rare, though less serious assaults and aggression are common [6]. Serious violence refers to the use of weapons, arson and assaults causing broken bones including life threatening acts such as homicide, stabbing, kidnap or penetrative sexual assault. The most serious act in this six month period was a physical assault on a nurse. There were 14 recorded acts of severe non-compliance with treatment, refusing to engage with treatment that led to a presentation of harmful behaviour or a serious deterioration in mental or physical condition (Table 3). In this paper the descriptions for each of these interventions have been reduced to a gradation from anticipation or reaction to behaviour up to placing restrictions or constraints on a patient. The majority of interventions were scored under situational coercion (*n* = 1994), which accounts for all patients being detained under mental health legislation. The more severe interventions were used less frequently. A restriction of privacy refers primarily to the patient being placed on enhanced observation levels, which was used on 401 patient-days but this observation was only increased to 2:1 at arm’s length on 16 occasions. The patients’ use of space was restricted on 380 days but 81% of those interventions were non-intrusive and resulted in the patient accessing quiet areas (*n* = 79) or being confined to a ward (*n* = 229). Seclusion was used for 39% of the patients (*n* = 11) and on 26 occasions on a total of 58 days (Table 4). Accordingly we may not have fully tested these ordinal scales at their extremes, even when observing over a six month period in an acute forensic admissions ward. However there is evidence in these findings that the scales are proportionate to each other over the ranges of behaviours and interventions most commonly observed.

The recording of daily routine DASA scores was sometimes patchy. There were 629 adverse incidents of varying degrees but only 178 DASA scores recorded above a zero, inclusive of 39 scores of four or above. The underreporting of lower levels of hostility, antisocial conduct and verbal aggression are well reported internationally [76] and this may in part explain this phenomenon.

We have not included women patients or adolescents in this study. This will require further studies.

We have not addressed milieu effects in this paper – this approach will form part of a second paper, however we note that this method readily lends itself to the study of milieu effects. Factors such as case mix, numbers of patients who are challenging on any given day, staff numbers (Table 2) and skills mix may all be in scope for a separate analysis using this paradigm.

We have not proceeded to examine whether interventions lead to reduced violence in this study. We believe this to be the case and will return to this with a more complex attempt at modelling lagged changes. Because these observations of necessity aggregate over a 24 h period, the period of time may be too long for a causal analysis of lagged change over hours or minutes. We believe that the use of closed circuit television recording (CCTV) will allow the future testing of causal models, along with real time rating of these ladders using hand held or similar electronic devices.

### Interpretation

At the individual level, a range of behaviours may be displayed by a disturbed and challenging patient. An analysis of just one form of behaviour such as violence, considered without regard to other behaviours such as placing others at risk, attempting to escape or abscond or non-compliance with treatment, and self-harm, would fail to capture the wholistic context and decision pathways that clinicians must weigh up and respond to in order to maintain a safe environment for the individual patient, for other patients and all who are present on the ward including visitors.

Similarly, an analysis of just one form of intervention, expressed as a binary outcome such as ‘seclusion’ or ‘manual restraint’ misses the complexity of how skilled clinicians deploy a range of interventions including de-escalation, observations, personal searches, extra medication, situational coercion, manual restraint, mechanical restraint and restricted use of space, with each of these being deployed along a gradation of seriousness according to the behaviours of the patient and the situation as it develops over minutes, hours or days.

It appears that staff exercise these interventions in a way that is proportionate to the seriousness of the behaviour. Since the behaviour is adumbrated by a risk measure made on the previous day, and this survives correction for repeated measures, the interventions appear to be used reasonably to de-escalate and prevent immediate and more serious violence. A gold standard would be the demonstration that subsequent violence was actually reduced. This paradigm will enable an analysis that could test this.

### Generalisability

The hospital care and treatment of people with severe mental disorders is increasingly limited to those who cannot be managed at home due to a need for intensive care to prevent suicide, severe self-harm and violence to others. An increasing proportion of those admitted to acute psychiatric units are detained under civil and forensic mental health legislation because of risk to themselves or others, while access to slow-stream rehabilitation and long-term psychiatric care is similarly related to forensic (court-mandated) pathways into care [77, 78].

The choice between the use of seclusion or restraint or extra medication and the most effective sequencing of interventions remains topical in the absence of research guidance.

We initially hypothesized that our range of risk assessment tools would identify those patients who would have adverse incidents and subsequently be subject to coercive interventions. We found little or no relationship between the HCR-20-C and subsequent behaviours or interventions.

We found small but significant associations between DASA scores on the previous day and the range of adverse incidents and interventions (Table 5). This may reflect on how well the DASA is scored and recorded by clinicians or may reflect the diversity and range of behaviours described under each ladder. For future research, an item-by item study of associations may be more informative, though this would require even larger sample sizes and longer periods of observation.

## Conclusions

As in the community, the emphasis in hospital care and treatment is on voluntarism and recovery, non-restraint and choice. There is a heightened awareness of the risks of perceived duress or implicit coercion, discrimination or disadvantage, amounting to structural violence [79–81]. However, suicide and violence in the context of severe mental disorders may at times require not only involuntary admission and detention, but also other intrusive, restrictive and coercive practices. Ethically, such manoeuvres are justified when medically necessary to save lives or to prevent serious harm. A question arises concerning the limits of medical necessity. In practice, in most jurisdictions, these interventions are permissible when authorised in accordance with law, when proportionate to need to prevent violence, when minimised in duration and when deployed where all other measures have failed [15].

The interventions used to prevent violence are notoriously lacking in the sort of scientific evidence required to meet the standards of Cochrane reviews or meta-analysis [3–5]. Interventions such as seclusion and restraint are commonly condemned from the point of view of principled human rights. It is not uncommon to hear of policy initiatives to completely eliminate the use of seclusion. At the same time it is universally acknowledged that violence should be completely prevented also. There is often little reflection on how these two principles can be reconciled. For example eliminating seclusion would probably lead to greatly increased use of prolonged manual restraint or mechanical restraint and forced medication by injection without consent [14]. Seclusion is probably safer than forced medication and seclusion is arguably more dignified than prolonged restraint. The lack of scientific evidence regarding the clinical use of restrictive, intrusive and coercive practices to prevent violence may now be addressed by means of this scientific nosology, which provides a valid rating system and the tools to carry out such studies.

We have observed some effects best explained by individual factors. Other effects may be best explained by milieu or systems factors for future research. We have established that disruptive and aggressive behaviours could be described as a range of behaviours that were coherent and internally consistent when rated and summated. We have also established that a range of interventions to prevent violence were deployed by staff in a way that was statistically proportionate, coherent and internally consistent. These observations are in keeping with a systems approach to understanding and improving outcomes [28]. In further research, we intend to address systems dynamic factors. We are conscious also that this observational study of antecedent risks, behaviours and interventions cannot examine the lagged causal models required to show that violence and other challenging behaviours is actually reduced by deploying this range of coordinated interventions. We intend to return to this also in future analysis and research. We hope that others will adopt this conceptual model and methodology which should eventually enable pragmatic randomised controlled trials.

Responding to high risk and violent behaviour by patients within a psychiatric setting always poses the problem of balancing the rights of the individual and the safety of others in the milieu. These practices all entail a loss of dignity, autonomy and self-determination on the part of the individual. Clinicians and institutions have a duty of care to provide a safe environment for all those present. In an unsafe environment, effective psychosocial interventions are impossible to sustain. There is an obligation to study how best to prevent or minimise violence by patients against others. This system of quantitative measures of behaviours and interventions is an attempt to provide the methods and measures for more systematic research regarding how to eliminate violence while at the same time minimising any actual or perceived institutional violence [75, 79–81]. It is now possible to study more complex treatment strategies.

These practices remain a reality of modern clinical practice. It is positive that they are reducing and, as this study has shown, are only used in proportion to the risk and actual behaviours presented, as a last resort and for as short a period of time as possible, even when caring for a population selected for serious violence in the context of severe mental disorders. We consider that the DRILL tool will be useful in daily practice for clinicians who are regularly required to demonstrate that forensic hospitals are safe and violence free, whilst also minimising the use of restrictive practices. We consider that the DRILL tool will support clinicians in both reviewing their own practices, those of their peers and also when demonstrating proportionality to outside reviewers such as CQC UK, MHC Ireland or Committee for Prevention of Torture (Europe).

## Supplementary information

**Additional file 1.**

## Data Availability

In view of the sensitive nature of the dataset concerning patients in a forensic hospital, this will not be made available however the dataset used and/or analysed during the current study is available from the corresponding author on reasonable request.

## Abbreviations

CCTV: Closed Circuit Television
CDP: Context Decision Path
CPT: The Council of Europe Committee for the Prevention of Cruel and Inhumane Treatment and Torture
CQC: Care Quality Commission UK
DASA: Dynamic Assessment of Situational Aggression
DRILL: Dundrum Restriction, Intrusion and Liberty Ladders
DRILL-B: DRILL Behaviour Ladders Score
DRILL-Int: DRILL Intervention Ladders Score
DUNDRUM-1: triage security scale
DUNDRUM-2: triage urgency scale
EssenCE: Essen Climate Evaluation Scale
FSS: Forensic Satisfaction Scale
GEE: General Estimating Equations
HCR-20: Historical, Clinical, Risk Management-20
HCR-20-C: Historical, Clinical, Risk Management-20 Clinical/Current items score
ICD-10: International Classification of Diseases 10th Edition
MHC: Mental Health Commission
PANSS: Positive and Negative Symptom Scale
PMVA: Prevention and Management of Violence and Aggression
QICC: Corrected Quasi Likelihood under Independence Model Criterion
RCI: Reliable Change Index
ROC-AUC: Receiver Operating Characteristic Area Under the Curve
SCID-1: Structured Clinical Interview for DSM-IV Axis I disorders
SMARG: Seclusion Monitoring and Restraint Group
SPSS-25: IBM Statistical Package for the Social Sciences 25
S-RAMM: Suicide Risk Assessment and Management Manual
