# Supplementary material for: A tool to evaluate proportionality and necessity in the use of restrictive practices in forensic mental health settings: the DRILL tool (Dundrum restriction, intrusion and liberty ladders)
Source: BMC Psychiatry. 2020 Oct 23;20:515. doi: 10.1186/s12888-020-02912-6 (PMC7583300; doi:10.1186/s12888-020-02912-6)
Supplement: Supplementary file 1 — Additional file 1. [file 12888_2020_2912_MOESM1_ESM.docx]

**Supplementary Table 1A:** **General Estimating Equation for effect of DASA previous day on DRILL Behaviour total score.**

| **DASA previous day** | **Beta** | **Wald X^2^** | **P** |
| --- | --- | --- | --- |
| **0** | **0.237** | **5.614** | **0.018** |
| **1** | **0.420** | **7.386** | **0.007** |
| **2** | **0.830** | **9.210** | **0.002** |
| **3** | **1.333** | **18.968** | **0.000** |
| **4** | **4.077** | **4.922** | **0.027** |
| **5** | **3.333** | **5.811** | **0.016** |
| **6** | **5.714** | **19.473** | **0.000** |
| **7** | **5.333** | **11.748** | **0.001** |

Goodness of fit: Ind: 2369.7; effect of DASA previous day Wald X^2^=146.7, df=8, p<0.001

**Supplementary Table 1B: General Estimating Equation for effect of DASA previous day on DRILL violent behaviour score.**

| **DASA previous day** | **Beta** | **Wald X^2^** | **p** |
| --- | --- | --- | --- |
| **0** | **0.033** | **3.164** | **0.075** |
| **1** | **0.012** | **1.589** | **0.208** |
| **2** | **0.109** | **7.070** | **0.008** |
| **3** | **0.143** | **4.009** | **0.045** |
| **4** | **0.500** | **9.000** | **0.003** |
| **5** | **0.444** | **4.662** | **0.031** |
| **6** | **0.714** | **5.619** | **0..018** |
| **7** | **0.667** | **4.174** | **0.041** |

**Goodness of fit: QICC = 122.2; Effects of DASA previous day X^2^=170.9, df=8, p<0.001**

**Supplementary Table 1C: General Estimating Equation for effect of DASA previous day on DRILL self-harming behaviour score.**

| **DASA previous day** | **Beta** | **Wald X^2^** | **P** |
| --- | --- | --- | --- |
| **0** | **0.049** | **2.874** | **0.090** |
| **1** | **1.049** | **0.931** | **0.335** |
| **2** | **0.000** | **-** | **-** |
| **3** | **0.095** | **0.930** | **0.335** |
| **4** | **0.154** | **1.174** | **0.279** |
| **5** | **0.111** | **1.191** | **0.275** |
| **6** | **0.429** | **2.205** | **0.138** |
| **7** | **0.000** | **-** | **-** |

**QICC = 146.1; effect of DASA previous day X^2^=4.6, df=8, p = 0.596.**

**Supplementary Table 1D: General Estimating Equation for effect of DASA previous day on DRILL ‘placing others at risk’ behaviour score.**

| **DASA previous day** | **Beta** | **Wald X^2^** | **P** |
| --- | --- | --- | --- |
| **0** | **0.112** | **4.302** | **0.038** |
| **1** | **0.198** | **4.788** | **0.029** |
| **2** | **0.319** | **15.926** | **0.000** |
| **3** | **0.476** | **12.872** | **0.000** |
| **4** | **0.923** | **9.310** | **0.002** |
| **5** | **1.111** | **13.729** | **0.000** |
| **6** | **1.429** | **24.500** | **0.000** |
| **7** | **1.167** | **24.000** | **0.000** |

**QICC = 370.54; effect of DASA previous day X^2^=97.3, df = 8, p <0.001**

**Supplementary Table 1E: General Estimating Equation for effect of DASA previous day on DRILL escape behaviour score.**

| **DASA previous day** | **Beta** | **Wald X^2^** | **P** |
| --- | --- | --- | --- |
| **0** | **0.008** | **2.204** | **0.138** |
| **1** | **0.000** | **-** | **-** |
| **2** | **0.021** | **1.022** | **0.312** |
| **3** | **0.048** | **1.021** | **0.312** |
| **4** | **0.692** | **1.432** | **0.231** |
| **5** | **0.333** | **0.942** | **0.332** |
| **6** | **0.429** | **1.114** | **0.291** |
| **7** | **0.917** | **2.434** | **0.119** |

**QICC = 91.2; effect of DASA previous day X^2^=33.9, df=8, p < 0.001**

**Supplementary Table 1F: General Estimating Equation for effect of DASA previous day on DRILL non-compliance behaviour score.**

| **DASA previous day** | **Beta** | **Wald X^2^** | **P** |
| --- | --- | --- | --- |
| **0** | **0.035** | **3.793** | **0.051** |
| **1** | **0.160** | **2.993** | **0.081** |
| **2** | **0.383** | **5.875** | **0.015** |
| **3** | **0..571** | **8.036** | **0.005** |
| **4** | **1.846** | **4.593** | **0.032** |
| **5** | **1.333** | **4.469** | **0.035** |
| **6** | **2.714** | **21.947** | **0.000** |
| **7** | **2.583** | **13.655** | **0.000** |

**QICC = 437.8; effect of DASA previous day X^2^=290.1, df= 8, p < 0.001**

**Supplementary Table 2A: General Estimating Equation for effect of DASA previous day on DRILL Intervention total score.**

| **DASA previous day** | **Beta** | **Wald X^2^** | **P** |
| --- | --- | --- | --- |
| **0** | **1.770** | **58.581** | **0.001** |
| **1** | **2.914** | **30.932** | **0.001** |
| **2** | **5.596** | **35.159** | **0.001** |
| **3** | **6.429** | **17.593** | **0.001** |
| **4** | **11.615** | **24.507** | **0.001** |
| **5** | **12.556** | **16.198** | **0.001** |
| **6** | **18.571** | **31.193** | **0.001** |
| **7** | **16.917** | **86.809** | **0.001** |

**QICC = 17891.017; effect of DASA previous day X^2^=1034.825, df= 8, p < 0.001**

**Supplementary Table 2B: General Estimating Equation for effect of DASA previous day on DRILL de-escalation score.**

| **DASA previous day** | **Beta** | **Wald X^2^** | **P** |
| --- | --- | --- | --- |
| **0** | **0.090** | **6.299** | **0.012** |
| **1** | **0.210** | **4.996** | **0.026** |
| **2** | **0.723** | **15.785** | **0.000** |
| **3** | **1.000** | **7.875** | **0.005** |
| **4** | **1.692** | **39.515** | **0.000** |
| **5** | **1.889** | **12.412** | **0.000** |
| **6** | **2.143** | **16.705** | **0.000** |
| **7** | **2.167** | **41.814** | **0.000** |

**QICC = 686.164; effect of DASA previous day X^2^=904.125, df= 8, p <0.000**

**Supplementary Table 2C: General Estimating Equation for effect of DASA previous day on DRILL observations score.**

| **DASA previous day** | **Beta** | **Wald X^2^** | **P** |
| --- | --- | --- | --- |
| **0** | **0.203** | **9.371** | **0.002** |
| **1** | **0.605** | **16.175** | **0.000** |
| **2** | **0915** | **35.413** | **0.000** |
| **3** | **1.095** | **35.347** | **0.000** |
| **4** | **2.231** | **9.283** | **0.000** |
| **5** | **2.000** | **16.200** | **0.000** |
| **6** | **2.714** | **13.220** | **0.000** |
| **7** | **2.333** | **10.138** | **0.001** |

**QICC =930.853; effect of DASA previous day X^2^ =119.967 , df= 8, p < 0.000**

**Supplementary Table 2D:** **General Estimating Equation for effect of DASA previous day on DRILL extra medication score.**

| **DASA previous day** | **Beta** | **Wald X^2^** | **P** |
| --- | --- | --- | --- |
| **0** | **0.015** | **5.047** | **0.025** |
| **1** | **0.025** | **1.080** | **0.299** |
| **2** | **0.170** | **6.139** | **0.013** |
| **3** | **0.524** | **5.393** | **0.020** |
| **4** | **0.154** | **1.178** | **0.279** |
| **5** | **1.000** | **5.786** | **0.016** |
| **6** | **1.857** | **25.559** | **0.000** |
| **7** | **1.000** | **6.000** | **0.014** |

**QICC =201.921; effect of DASA previous day X^2^=103.136, df= 8, p <0.000**

**Supplementary Table 2E: General Estimating Equation for effect of DASA previous day on DRILL physical (manual) restraint score.**

| **DASA previous day** | **Beta** | **Wald X^2^** | **P** |
| --- | --- | --- | --- |
| **0** | **0.011** | **3.333** | **0.068** |
| **1** | **0.000** |  |  |
| **2** | **0.170** | **5.133** | **0.023** |
| **3** | **0.286** | **1.670** | **0.196** |
| **4** | **0.000** |  |  |
| **5** | **0.667** | **3.115** | **0.078** |
| **6** | **1.143** | **2.513** | **0.113** |
| **7** | **1.000** | **3.789** | **0.052** |

**QICC =197.536; effect of DASA previous day X^2^=44.992, df= 8, p <0.000**

**Supplementary Table 2F: General Estimating Equation for effect of DASA previous day on DRILL seclusion score.**

| **DASA previous day** | **Beta** | **Wald X^2^** | **P** |
| --- | --- | --- | --- |
| **0** | **0.326** | **8.508** | **0.004** |
| **1** | **0.716** | **8.209** | **0.004** |
| **2** | **1.404** | **13.087** | **0.000** |
| **3** | **1.286** | **7.988** | **0.005** |
| **4** | **3.000** | **17.284** | **0.000** |
| **5** | **3.000** | **52.071** | **0.000** |
| **6** | **4.571** | **185.837** | **0.000** |
| **7** | **4.250** | **127.656** | **0.000** |

**QICC =1952.499; effect of DASA previous day X^2^ =2847.712, df= 8, p <0.000**

**Supplementary Table 2G: General Estimating Equation for effect of DASA previous day on DRILL searches score.**

| **DASA previous day** | **Beta** | **Wald X^2^** | **P** |
| --- | --- | --- | --- |
| **0** | **0.053** | **6.397** | **0.011** |
| **1** | **0.148** | **5.106** | **0.024** |
| **2** | **0.511** | **5.259** | **0.022** |
| **3** | **0.429** | **2.901** | **0.089** |
| **4** | **1.385** | **7.456** | **0.006** |
| **5** | **1.333** | **6.291** | **0.012** |
| **6** | **2.571** | **58.800** | **0.000** |
| **7** | **2.250** | **58.909** | **0.000** |

**QICC =514.677; effect of DASA previous day X^2^ =1115.610, df= 8, p <0.000**

**Supplementary Table 2H: General Estimating Equation for effect of DASA previous day on DRILL situational coercion score.**

| **DASA previous day** | **Beta** | **Wald X^2^** | **P** |
| --- | --- | --- | --- |
| **0** | **1.072** | **1220.822** | **0.000** |
| **1** | **1.210** | **109.978** | **0.000** |
| **2** | **1.702** | **65.052** | **0.000** |
| **3** | **1.810** | **29.295** | **0.000** |
| **4** | **30.154** | **40.747** | **0.000** |
| **5** | **2.667** | **16.831** | **0.000** |
| **6** | **3.571** | **18.975** | **0.000** |
| **7** | **3.917** | **94.503** | **0.000** |

**QICC =794.619; effect of DASA previous day X^2^ =2167.617, df= 8, p <0.000**

**Supplementary Table 3A: General Estimating Equation for effect of DRILL Behaviours (0-7) total score on DRILL Interventions total score.**

| **DASA previous day** | **Beta** | **Wald X^2^** | **P** |
| --- | --- | --- | --- |
| **0** | **1.373** | **106.919** | **0.000** |
| **1** | **3.285** | **53.075** | **0.000** |
| **2** | **9.500** | **31.980** | **0.000** |
| **3** | **12.522** | **126.556** | **0.000** |
| **4** | **8.269** | **6.818** | **0.000** |
| **5** | **15.714** | **251.395** | **0.000** |
| **6** | **20.100** | **1134.222** | **0.000** |
| **7** | **23.500** | **440.576** | **0.000** |

**QICC =8291.527; effect of DRILL Behaviours (0-7) total score Wald X^2^ =9413.043, df= 8, p=0.000**

**Supplementary Table 3B: General Estimating Equation for effect of DRILL Behaviours (0-7) total score on DRILL de-escalation score.**

| **DRILL Behaviours total (0-7)** | **Beta** | **Wald X^2^** | **P** |
| --- | --- | --- | --- |
| **0.0000** | **0.009** | **5.333** | **0.021** |
| **1** | **0.205** | **2.088** | **0.148** |
| **2** | **1.429** | **25.960** | **0.000** |
| **3** | **2.087** | **42.279** | **0.000** |
| **4** | **1.692** | **10.557** | **0.001** |
| **5** | **2.333** | **8.467** | **0.004** |
| **6** | **3.700** | **3602.632** | **0.000** |
| **7** | **3.000** | **72.000** | **0.000** |

**QICC =282.009; effect of DRILL Behaviours (0-7) total score Wald X^2^ =5654.789, df= 8, p=0.000**

**Supplementary Table 3C: General Estimating Equation for effect of DRILL Behaviours (0-7) total score on DRILL observations score.**

| **DRILL Behaviours total (0-7)** | **Beta** | **Wald X^2^** | **P** |
| --- | --- | --- | --- |
| **0** | **0.138** | **7.759** | **0.005** |
| **1** | **0.921** | **48.277** | **0.000** |
| **2** | **.071** | **170.600** | **0.000** |
| **3** | **1.696** | **8.631** | **0.003** |
| **4** | **0.519** | **1.802** | **0.179** |
| **5** | **3.619** | **108.346** | **0.000** |
| **6** | **2.000** | **-** | **0.000** |
| **7** | **4.500** | **72.000** | **0.000** |

**QICC =646.025; effect of DRILL Behaviours (0-7) total score Wald X^2^ =1426.940, df=7, p=0.000**

**Supplementary Table 3D: General Estimating Equation for effect of DRILL Behaviours (0-7) total score on DRILL Extra Medication score.**

| **DRILL Behaviours total (0-7)** | **Beta** | **Wald X^2^** | **P** |
| --- | --- | --- | --- |
| **0** | **0.003** | **1.986** | **0.159** |
| **1** | **0.020** | **0.831** | **0.362** |
| **2** | **0.179** | **1.003** | **0.317** |
| **3** | **0.522** | **9.301** | **0.002** |
| **4** | **0.442** | **1.706** | **0.192** |
| **5** | **0.762** | **5.485** | **0.019** |
| **6** | **1.000** | **25.000** | **0.000** |
| **7** | **1.750** | **15.680** | **0.000** |

**QICC =200.660; effect of DRILL Behaviours (0-7) total score Wald X^2^ =428.583, df=8, p=0.000**

**Supplementary Table 3E: General Estimating Equation for effect of DRILL Behaviours (0-7) total score on DRILL Physical (Manual) Restraint score.**

| **DRILL Behaviours total (0-7)** | **Beta** | **Wald X^2^** | **P** |
| --- | --- | --- | --- |
| **0** | **0.000** | **-** | **-** |
| **1** | **0.013** | **0.831** | **0.362** |
| **2** | **0.071** | **1.003** | **0.317** |
| **3** | **0.087** | **1.254** | **0.263** |
| **4** | **0.327** | **1.154** | **0.282** |
| **5** | **0.143** | **0.823** | **0.364** |
| **6** | **2.100** | **53.650** | **0.000** |
| **7** | **1.250** | **2.140** | **0.143** |

**QICC =153.820; effect of DRILL Behaviours (0-7) total score Wald X^2^ =478.154, df=7, p=0.000**

**Supplementary Table 3F: General Estimating Equation for effect of DRILL Behaviours (0-7) total score on DRILL Seclusion score.**

| **DRILL Behaviours total (0-7)** | **Beta** | **Wald X^2^** | **P** |
| --- | --- | --- | --- |
| **0** | **0.196** | **5.911** | **0.015** |
| **1** | **0.901** | **4.250** | **0.039** |
| **2** | **2.393** | **32.996** | **0.000** |
| **3** | **3.783** | **77.832** | **0.000** |
| **4** | **2.692** | **27.950** | **0.000** |
| **5** | **3.429** | **23.607** | **0.000** |
| **6** | **4.600** | **601.136** | **0.000** |
| **7** | **5.000** | **-** | **0.000** |

**QICC =1202.687; effect of DRILL Behaviours (0-7) total score Wald X^2^ =950.504, df=7, p=0.000**

**Supplementary Table 3G: General Estimating Equation for effect of DRILL Behaviours (0-7) total score on DRILL Searches score.**

| **DRILL Behaviours total (0-7)** | **Beta** | **Wald X^2^** | **P** |
| --- | --- | --- | --- |
| **0** | **0.015** | **1.151** | **0.283** |
| **1** | **0.139** | **2.465** | **0.116** |
| **2** | **1.821** | **7.422** | **0.006** |
| **3** | **1.174** | **3.946** | **0.047** |
| **4** | **0.692** | **1.848** | **0.174** |
| **5** | **1.000** | **4.793** | **0.029** |
| **6** | **2.100** | **50.000** | **0.000** |
| **7** | **3.000** | **-** | **0.000** |

**QICC =414.021; effect of DRILL Behaviours (0-7) total score Wald X^2^ =433.742, df=7, p=0.000**

**Supplementary Table 3H: General Estimating Equation for effect of DRILL Behaviours (0-7) total score on DRILL situational coercion score.**

| **DRILL Behaviours total (0-7)** | **Beta** | **Wald X^2^** | **P** |
| --- | --- | --- | --- |
| **0** | **1.012** | **22408.727** | **0.000** |
| **1** | **1.086** | **283.250** | **0.000** |
| **2** | **2.536** | **14.321** | **0.000** |
| **3** | **3.174** | **62.167** | **0.000** |
| **4** | **1.904** | **8.008** | **0.000** |
| **5** | **4.429** | **203.750** | **0.000** |
| **6** | **4.600** | **2938.889** | **0.000** |
| **7** | **5.000** | **-** | **0.000** |

**QICC =396.930; effect of DRILL Behaviours (0-7) total score Wald X^2^ =70999.283, df=7, p=0.000**
